# Associations between *CYP1A1* rs1048943 A > G and rs4646903 T > C genetic variations and colorectal cancer risk: Proof from 26 case-control studies

**DOI:** 10.18632/oncotarget.10331

**Published:** 2016-06-30

**Authors:** Xueru Zhu, Zhao Wang, Jing He, Weiye Wang, Wenji Xue, Yiwei Wang, Leizhen Zheng, Mei-Ling Zhu

**Affiliations:** ^1^ Department of Oncology, Xin Hua Hospital Affiliated to Shanghai Jiaotong University School of Medicine, Yangpu, Shanghai, China; ^2^ Sun Yat-Sen University Cancer Center, State Key Laboratory of Oncology in South China, Department of Experimental Research, Collaborative Innovation Center for Cancer Medicine, Guangzhou, Guangdong, China; ^3^ Department of Pediatric Surgery, Guangzhou Institute of Pediatrics, Guangzhou Women and Children's Medical Center, Guangzhou Medical University, Guangzhou, Guangdong, China; ^4^ Shanghai Key Laboratory of Children's Environmental Health, Xin Hua Hospital Affiliated to Shanghai Jiaotong University School of Medicine, Yangpu, Shanghai, China

**Keywords:** CYP1A1, polymorphism, colorectal cancer, meta-analysis

## Abstract

Cytochrome P450 1A1 (CYP1A1) enzyme is one of the most important metabolizing enzymes responsible for the metabolism of numerous xenobiotics. Numerous individual case-control studies have investigated the associations between the *CYP1A1* rs1048943 A > G and rs4646903 T > C genetic variations and colorectal cancer (CRC) risk, but the conclusions were controversial. To obtain a scientific conclusion, we performed a meta-analysis based on a total of 26 publications, including 20 studies with 8665 cases and 9953 controls on rs1048943 A > G and 19 studies with 6416 cases and 7551 controls on rs4646903 T > C, respectively. The pooled analysis indicated that rs1048943 A > G was associated with an increased risk of CRC (G vs. A: OR = 1.28, 95% CI = 1.08−1.52; GG vs. AA: OR = 1.54, 95% CI = 1.25−1.91; GA vs. AA: OR = 1.26, 95% CI = 1.00−1.60; GG/GA vs. AA: OR = 1.31, 95% CI = 1.05−1.64; GG vs. GA/AA: OR = 1.56, 95% CI = 1.26−1.91). Stratification analysis showed the association between rs1048943 A > G and CRC risk was more obvious in studies with the population-based (PB) design or high quality score. The association between rs4646903 T > C and CRC risk did not reach statistical significance in the pooled analysis as well as stratification analysis. This meta-analysis demonstrated *CYP1A1* rs1048943 A > G may increase the susceptibility to CRC instead of rs4646903 T > C. This conclusion suggested *CYP1A1* may contribute to the pathogenesis of CRC.

## INTRODUCTION

Although the incidence rate of CRC decreased by approximately 3% per year during the past decade, CRC is still one of the most common cancers and the third leading cause of cancer-related death worldwide [1]. Despite the fact of great improvement in chemotherapy and surgical operations, the prognosis of advanced CRC is still much worse than early-stage CRC [2]. Therefore, for cancer prevention and early diagnosis, it is important to identify risk factors and biomarkers that are associated with disease susceptibility to screen high risk population.

The development of CRC is widely considered as a multi-step, multi-factorial process involving gene-gene and gene-environment interactions [3]. Previous studies have shown that lifestyle factors, such as cigarette smoking and alcohol consumption may contribute to sporadic CRC risk [4]. *In vivo*, these xenobiotics (e.g. nicotine and alcohol) are metabolized by xenobiotic-metabolizing enzymes including CYP1A1 superfamily, glutathione S-transferases, N-acetyltransferase, etc [5]. Furthermore, genetic variations of these enzymes may lead to the occurrence of CRC by metabolizing environmental insults [3]. In recent years, an increasing number of individual case-control studies have investigated the association of genetic variations within cytochrome P450s (CYPs) with CRC risk.

The Human Genome Project has identified 57 human cytochrome P450 enzymes, and ordered the minto 18 families and 43 subfamilies by sequence similarities [6]. Although most chemical carcinogens are inactive *in vivo*, they can become bio-active via CYPs. For example, benzo[a] pyrene can be metabolized and transformed to mutagenic benzo[a] pyrene diol epoxide [7, 8]. CYPs, the key of phase I enzymes, are the main enzymes in the metabolism of carcinogenic polycyclic aromatic hydrocarbons (PAHs) [9]. Among all researches of *CYP* involvement in procarcinogen activation, *CYP1A1s* polymorphisms of rs1048943 A > G and rs4646903 T > C were the most widely studied [10]. *CYP1A1* rs1048943 A > G leads to amino acid change in exon 7 of *CYP1A1* from Ile to Val (nucleotides A to G) at codon 462. *CYP1A1* rs4646903 T > C is characterized by the T to C mutation at nucleotide 3801 in the 3′-flanking region of the gene [11].

To date, numerous case-control studies have investigated the associations between *CYP1A1* rs1048943 A > G, rs4646903 T > C and CRC, but the conclusions were inconsistent. Hence, we conducted a meta-analysis to obtain a scientific conclusion.

## RESULTS

### Characteristics of eligible publications

**Figure 1:**
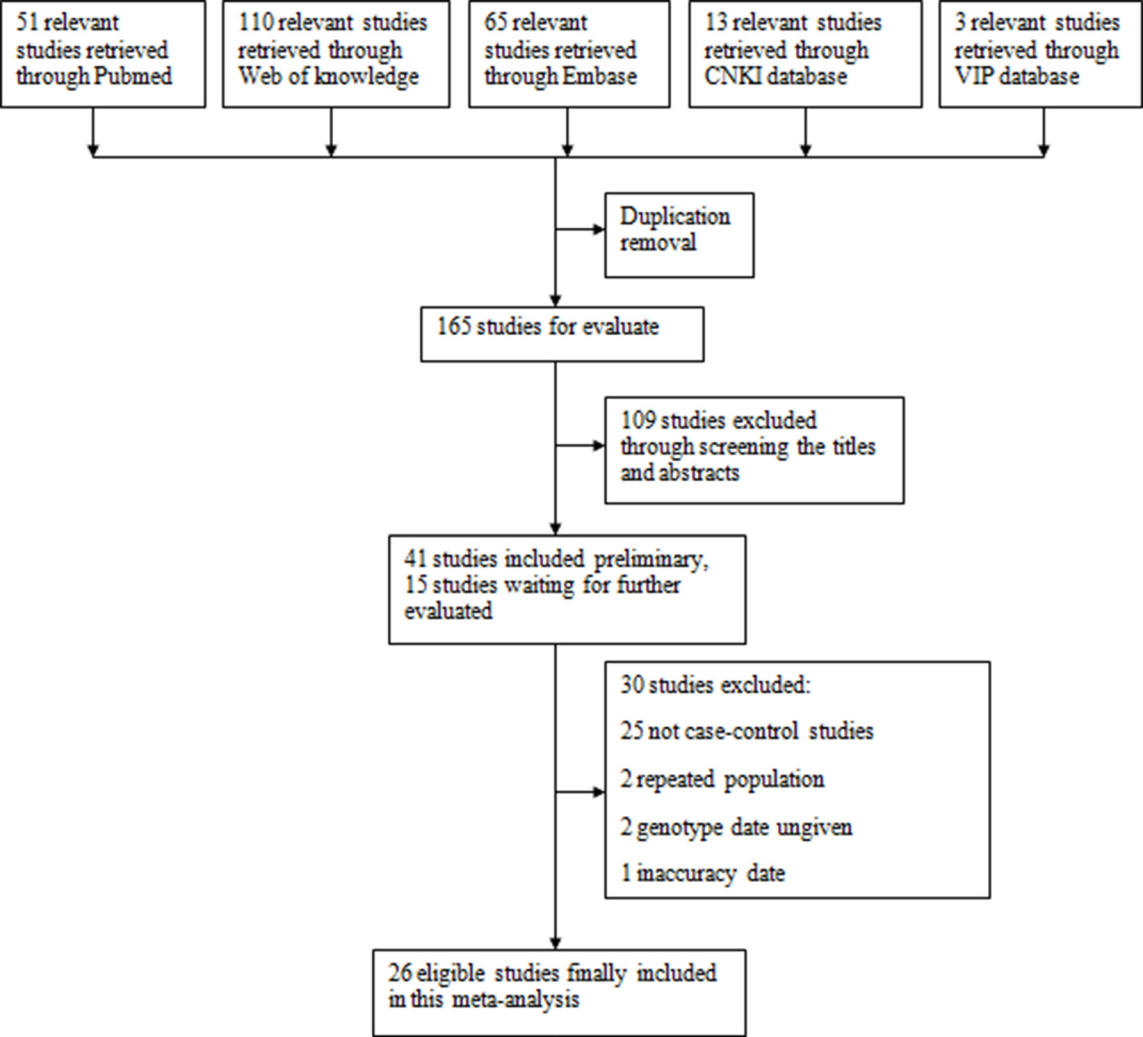
Flow chart of studies selection

A total of 26 articles reporting the associations between *CYP1A1* polymorphisms and CRC risk were included in the meta-analysis. There were 20 studies (8665 cases and 9953 controls) for rs1046943 A > G and 19 studies (6416 cases and 7551 controls) for rs4646903 T > C. The study selection process was shown in Figure 1 [5, 11–35].

The ethnicities of the 20 publications about rs1048943 A > G, included Asian (6 studies), Caucasian (13 studies) and mixed populations (1 study). The distribution of genotype among controls fulfilled the criteria of HWE in14 studies. There were 18 studies with high quality scores. For rs4646903 T > C, the ethnicities of the 19 publications included Asian (7 studies) and Caucasian (12 studies). The distribution of genotype among controls fulfilled the criteria of HWE in 17 studies. There were 17 studies with high quality scores. The main characteristics of the 26 eligible studies were listed in Table 1.

**Table 1:** Characteristics of the 26 articles included in the meta-analysis

Study	Country	Ethnicity	Source	Polymorphism	Sample size	HWE	HWE	Score
			of control	type	(case/control)	(cases)	(control)	
Hayashi,1992	Japan	Asian	PB	rs1048943	85/358	0.114	0.332	12
Sivaraman,1994	Mixed	Caucasian	PB	rs1048943	43/47	0.227	0.230	11
				rs4646903	43/47	0.001	0.245	11
Frische,1999	Germany	Caucasian	PB	rs1048943	187/101	0.622	0.839	13
				rs4646903	187/101	0.496	0.563	13
Ishibe,2000	America	Caucasian	PB	rs1048943	212/221	0.018	0.057	13
				rs4646903	212/221	0.247	0.428	13
Sachse,2002	Britain	Caucasian	PB	rs1048943	490/592	0.035	0.002	11
				rs4646903	490/592	0.040	0.596	14
Ye,2002	Britain	Caucasian	PB	rs4646903	41/82	0.613	0.599	11
Slattery,2004	America	Caucasian	PB	rs1048943	1791/2180	0.000	0.000	12
				rs4646903	1805/2164	0.080	0.118	15
Hou,2005	Mixed	Mixed	HB	rs1048943	675/679	0.117	0.478	12
Landi,2005	Spain	Caucasian	HB	rs1048943	362/323	0.617	0.469	11
				rs4646903	358/305	0.717	0.793	11
Chen,2005	China	Asian	PB	rs4646903	139/340	0.978	0.821	13
Little,2006	Britain	Caucasian	PB	rs1048943	251/396	0.602	0.534	14
				rs4646903	232/378	0.206	0.448	14
Bente,2006	Australia+ Poland	Caucasian	HB	rs4646903	118/100	0.039	0.637	10
Youshida,2007	Japan	Asian	PB	rs1048943	66/121	0.910	0.800	11
				rs4646903	66/121	0.244	0.623	11
Yeh,2007	Taiwan, China	Asian	HB	rs1048943	717/729	0.000	0.280	12
Kiss,2007	Hungary	Caucasian	HB	rs1048943	500/500	0.172	0.315	12
Pereira, 2008	Brazil	Caucasian	PB	rs1048943	114/114	0.000	0.071	12
Pande,2008	America	Caucasian	PB	rs1048943	120/137	0.294	0.000	10
				rs4646903	120/137	0.046	0.272	13
Zheng,2009	China	Asian	PB	rs1048943	79/110	0.056	0.031	8
				rs4646903	79/110	0.479	0.272	11
Liu,2009	China	Asian	HB	rs4646903	75/100	0.000	0.000	6
Kobayashi,2009	Japan	Asian	HB	rs1048943	117/289	0.164	0.000	7
Nisa,2010	Japan	Asian	PB	rs1048943	685/778	0.580	0.970	14
				rs4646903	685/778	0.423	0.718	14
Cleary,2010	Canada	Caucasian	PB	rs1048943	1174/1293	0.000	0.006	11
				rs4646903	1174/1293	0.281	0.062	14
Houlle,2011	France	Caucasian	HB	rs1048943	329/419	0.651	0.546	11
				rs4646903	329/419	0.804	0.362	11
Darazy,2011	Lebanon	Asian	PB	rs4646903	70/70	0.000	0.000	5
Saeed,2013	Saudi Arabia	Asian	HB	rs4646903	100/79	0.374	0.726	10
Gil,2014	Poland	Caucasian	HB	rs1048943	478/404	0.574	0.095	11

PB, population-based; HB, hospital-based; HWE, Hardy-Weinberg equilibrium.

### Meta-analysis

### *CYP1A1* rs1048943 A > G polymorphism

**Figure 2:**
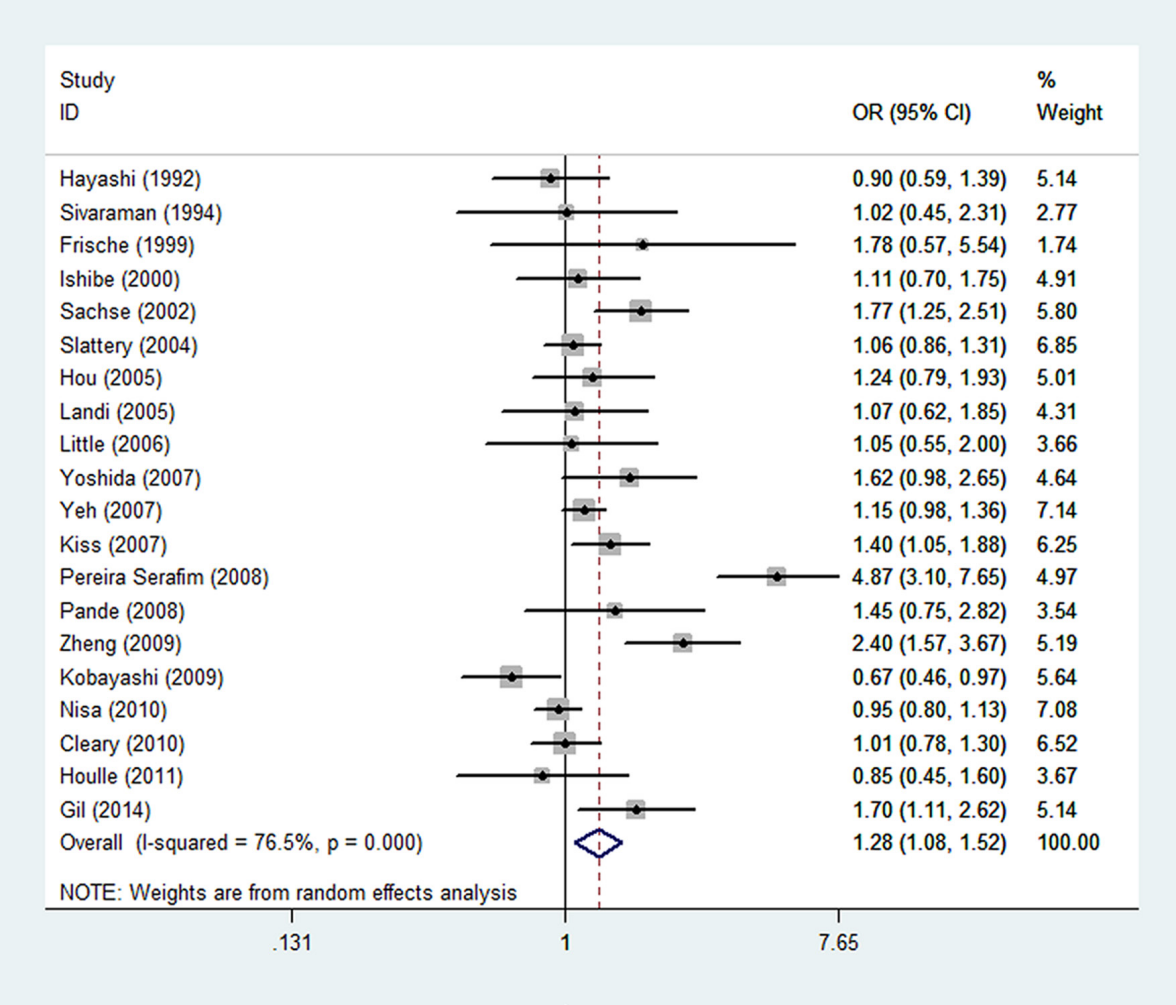
Forest plot of colorectal cancer risk associated with *CYP1A1* rs1048943 A>G polymorphism (G vs. A)

The association between *CYP1A1* rs1048943 A > G and the risk of CRC was shown in Table 2 and Figure 2. To sum up, the associations under all genetic models were statistically significant (G vs. A: OR = 1.28, 95% CI = 1.08–1.52; GG vs. AA: OR = 1.54, 95% CI = 1.25–1.91; GA vs. AA: OR = 1.26,95% CI = 1.00–1.60; GG/GA vs. AA: OR = 1.31, 95% CI = 1.05–1.64; GG vs. GA/AA: OR = 1.56, 95% CI = 1.26-1.91) (Supplementary Figure 1). Further stratified analysis showed the association was especially obvious in studies with population-based (PB) designs and of high quality scores. In studies of PB designs, the association was more obvious in the homozygous model (GG vs. AA: OR = 1.61, 95% CI = 1.03–2.53). In studies of high quality score, the association was more obvious using recessive model (GG vs. GA/AA: OR = 1.45, 95% CI = 1.16–1.82). Stratification analysis by ethnicity showed the association of rs1048943 A > G and CRC were more obvious in homozygous (GG vs. AA: OR = 1.61, 95% CI = 1.04–2.47) and recessive (GG vs. GA/AA: OR = 1.59, 95% CI = 1.25-2.02) models in Asian. In Caucasian, the associations were obvious in dominant model (GA/GG vs. AA: OR=1.43, 95% CI = 1.05–1.95), heterozygous model (GA vs. AA: OR = 1.42, 95% CI = 1.04–1.96) and G allelic (G vs. A: OR = 1.33, 95% CI = 1.06–1.68). After excluding studies whose distribution of genotype in controls deviated from HWE, the association remained statistically significant (G vs. A: OR = 1.30, 95% CI = 1.05–1.61; GG vs. AA: OR = 1.51, 95% CI = 1.17–1.94; GG/GA vs. AA: OR = 1.35, 95% CI = 1.01–1.81; GG vs. GA/AA: OR = 1.52, 95% CI = 1.19–1.95). All of the results were listed in Table 2.

**Table 2:** Meta-analysis of the association between *CYP1A1* polymorphisms and CRC risk

	G vs. A			GG vs. AA			GA vs. AA			GA/GG vs. AA			GG vs. GA/AA		
Variables	OR(95%CI)	*P*_OR^a^_	*P*_het^b^_	OR (95% CI)	*P*_OR^a^_	*P*_het^b^_	OR (95% CI)	*P*_OR^a^_	*P*_het^b^_	OR (95% CI)	*P*_OR^a^_	*P*_het^b^_	OR (95% CI)	*P*_OR^a^_	*P*_het^b^_
rs1048943	1.28 (1.08–1.52)	0.004	0.000	1.54 (1.25–1.91)	0.000	0.228	1.26 (1.00–1.60)	0.05	0.000	1.31 (1.05–1.64)	0.018	0.000	1.56 (1.26–1.91)	0.000	0.664
Ethnicity															
Asian	1.14 (0.87–1.50)	0.346	0.000	1.61 (1.04–2.47)	0.031	0.049	0.93 (0.67–1.29)	0.661	0.001	1.04 (0.75–1.48)	0.809	0.000	1.59 (1.25–2.02)	0.000	0.184
Caucasian	1.33 (1.06–1.68)	0.015	0.000	1.48 (0.95–2.32)	0.083	0.526	1.42 (1.04–1.96)	0.030	0.000	1.43 (1.05–1.95)	0.022	0.000	1.41 (0.92–2.17)	0.113	0.849
Source of control															
PB	1.40 (1.09–1.80)	0.009	0.000	1.61 (1.03–2.53)	0.038	0.054	1.48 (1.06–2.06)	0.021	0.000	1.52 (1.10–2.11)	0.011	0.000	1.37 (1.04–1.81)	0.024	0.373
HB	1.13 (0.91–1.40)	0.255	0.026	1.68 (1.22–2.33)	0.002	0.935	0.99 (0.72–1.38)	0.972	0.000	1.05 (0.79–1.41)	0.727	0.001	1.82 (1.33–2.50)	0.000	0.985
Score															
High	1.28 (1.08–1.52)	0.004	0.000	1.48 (1.18–1.86)	0.001	0.591	1.28 (1.01–1.62)	0.043	0.000	1.32 (1.05–1.65)	0.017	0.000	1.49 (1.19–1.86)	0.001	0.869
Low	1.32 (0.56–3.11)	0.530	0.000	1.57 (0.42–5.93)	0.507	0.030	1.24 (0.36–4.24)	0.735	0.000	1.31 (0.41–4.22)	0.652	0.000	2.02 (1.19–3.44)	0.009	0.141
HWE															
Yes	1.30 (1.05–1.61)	0.015	0.000	1.51 (1.17–1.94)	0.001	0.382	1.31 (0.96–1.77)	0.088	0.000	1.35 (1.01–1.81)	0.041	0.000	1.52 (1.19–1.95)	0.001	0.719
No	1.25 (0.91–1.73)	0.173	0.000	1.63 (1.11–2.41)	0.013	0.113	1.19 (0.79–1.79)	0.417	0.000	1.23 (0.83–1.83)	0.297	0.000	1.63 (1.12–2.39)	0.011	0.322
	C vs. T			CC vs. TT			CT vs. TT			CT/CC vs. TT			CC vs. CT/TT		
rs4646903	1.09 (0.96–1.23)	0.186	0.001	1.11 (0.81–1.53)	0.507	0.032	1.04 (0.91–1.18)	0.594	0.034	1.07 (0.93–1.22)	0.342	0.009	1.10 (0.82–1.47)	0.530	0.053
Ethnicity															
Asian	1.27 (0.92–1.75)	0.140	0.000	1.17 (0.75–1.82)	0.502	0.077	1.23 (0.85–1.77)	0.267	0.016	1.32 (0.89–1.96)	0.168	0.001	1.05 (0.84–1.32)	0.666	0.279
Caucasian	1.09 (0.92–1.28)	0.331	0.075	1.23 (0.89–1.72)	0.213	0.136	0.98 (0.88–1.10)	0.710	0.114	1.00 (0.90–1.12)	0.943	0.150	1.52 (0.81–2.85)	0.192	0.085
Source of control															
PB	0.99 (0.92–1.06)	0.774	0.193	1.04 (0.75–1.45)	0.826	0.058	0.98 (0.90–1.08)	0.726	0.175	0.99 (0.91–1.07)	0.745	0.235	1.05 (0.76–1.44)	0.783	0.064
HB	1.55 (0.92–2.61)	0.100	0.000	1.61 (0.92–2.82)	0.096	0.141	1.36 (0.86–2.14)	0.190	0.017	1.51 (0.90–2.54)	0.122	0.001	1.51 (0.87–2.62)	0.144	0.206
Score															
High	1.05 (0.93–1.18)	0.465	0.005	1.02 (0.74–1.41)	0.886	0.052	1.02 (0.90–1.17)	0.741	0.029	1.04 (0.91–1.18)	0.604	0.016	1.03 (0.76–1.40)	0.859	0.064
Low	1.85 (1.19–2.87)	0.006	0.644	2.24 (1.04–4.80)	0.039	0.905	1.62 (0.80–3.28)	0.180	0.696	1.88 (1.06–3.35)	0.031	0.709	1.99 (0.95–4.16)	0.068	0.846
HWE															
Yes	1.05 (0.93–1.18)	0.465	0.005	1.02 (0.74–1.41)	0.886	0.052	1.02 (0.90–1.17)	0.741	0.029	1.04 (0.91–1.18)	0.604	0.016	1.03 (0.76–1.40)	0.859	0.064
No	1.85 (1.19–2.87)	0.006	0.644	2.24 (1.04–4.80)	0.039	0.905	1.62 (0.80–3.28)	0.180	0.696	1.88 (1.06–3.35)	0.031	0.709	1.99 (0.95–4.16)	0.068	0.846

a *P* value of the *Z*-test for odds ratio test;

b *P* value of the *Q*-test for heterogeneity test; PB, population based; HB, hospital based; HWE, Hardy-Weinberg equilibrium.

### *CYP1A1* rs4646903 T > C polymorphism

In the meta-analysis, the pooled analysis showed no significant association between rs4646903 T > C and CRC risk. Further stratification analyses also found no obvious association except the subgroups of deviating from HWE and low quality score which included the same two studies showed statistical significances (C vs. T: OR = 1.85, 95% CI = 1.19–2.87; CC vs. TT: OR = 2.24, 95% CI = 1.04–4.80; CT/CC vs. TT: OR = 1.88, 95% CI = 1.06–3.35) (Table 2).

### Heterogeneity and sensitivity analysis

The Chi-squared-based Q-test showed substantial heterogeneities among publications for the two polymorphisms (Table 2). Meta-regression analysis for both polymorphisms yielded no significant difference between subgroups, except subgroups of HWE and study quality score for rs464903 T > C. After excluding the inferior quality studies of deviate from HWE and low score, the conclusions of both polymorphisms were not change. Furthermore, we estimated the influence of single individual data on the combined ORs by consecutively omitting each study from the meta-analysis, no obvious differences were observed for both variations (Supplementary Figure 2).

### Publication bias

**Figure 3:**
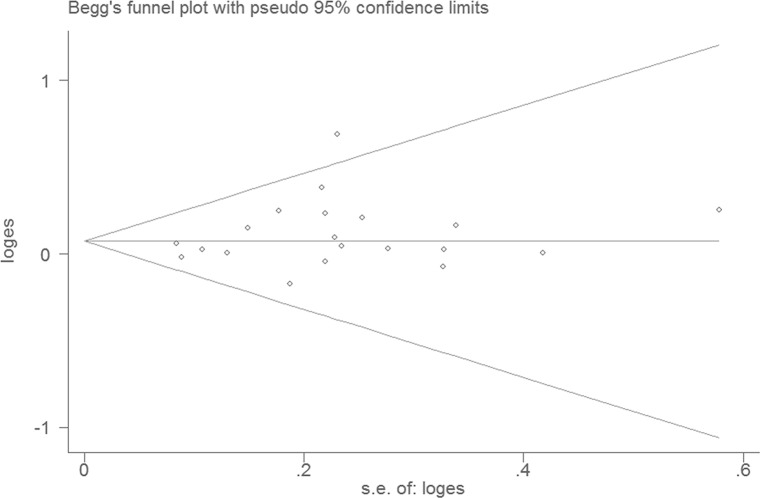
Begg's funnel plot of colorectal cancer risk associated with *CYP1A1* rs1048943 A >G polymorphism (G vs. A)

For rs1048943 A > G, the shapes of funnel plots were symmetric (Figure 3) and Egger's linear regression test provided no evidence of publication bias (G vs. A: *P* = 0.185, *t* = 1.38; GG vs. AA: *P* = 0.325, *t* = 1.01; GA vs. AA: *P* = 0.132, *t* = 1.58; GG/GA vs. AA: *P* = 0.116, *t* = 1.65; GG vs. GA/AA: *P* = 0.587, *t* = 0.56). For rs4646903 T > C the funnel plots were also symmetric and Egger's linear regression test provided no evidence of publication bias (CC vs. TT: *P* = 0.174, *t* = 1.42; CT vs. TT: *P* = 0.056, t = 2.05; CC vs. CT/TT: *P* = 0.234, *t* = 1.24), except two models of G allelic (C vs. T: *P* = 0.005, *t* = 3.27) and dominant model (CT/CC vs. TT: *P* = 0.013, *t* = 2.78).

## DISCUSSION

In this meta-analyses, we comprehensively evaluated the associations between two polymorphisms (rs1048943 A > G and rs4646903 T > C) and CRC risk through 26 studies. We observed that rs1048943 A > G genetic variation was associated with increased risk of CRC. The association remained statistically significant in subgroups (Asians, Caucasians, PB, high quality score and the studies consistent with HWE). On the contrary, the association between rs4646903 T > C and CRC risk did not reach the significance level.

CYP1A1 protein is a member of CYP superfamily and widely distributes in lung, kidney, intestine, skin, larynx, placenta, lymphocyte, brain tissues [36]. Because the main role of this enzyme is to metabolize environmental carcinogens, such as PAHs, heterocyclic amines, aflatoxin B1 and estrogen [37], variations within *CYP1A1* gene may induce the occurrence of CRC. Currently, a widely accepted paradigm for CYP1A1 enzyme mediated carcinogens activation is that CYP1A1 metabolizes polycyclic aromatic hydrocarbons to reactive epoxide intermediates, which could covalently bind to DNA and then induce tumors [38].

Due to the high minor allele frequency (MAF) as well as their possible involvement in an increased risk of several carcinomas [39], including lung cancer, colorectal cancer, breast cancer, leukemia, esophageal carcinoma and prostate cancer [40], *CYP1A1* rs1048943 A > G and rs4646903 T > C polymorphisms are more widely studied. In addition to inducing the occurrence of cancers, the polymorphisms of *CYP1A1* may also lead to other diseases, such as ulcerative colitis, colorectal adenoma, atherosclerosis, myocardial infarction and so on [41–43].

Partial meta-analyses on the association of *CYP1A1* polymorphisms and colorectal cancer risk showed that *CYP1A1* rs1048943 A > G might be associated with increased risk of CRC [44, 45]. In the present study, we combined the studies to increase sample size and further validated this results. However, contradictory results were observed in a previous meta-analysis which concluded that there was no association between rs1048943 A > G and CRC risk [46]. This discrepancy mainly results from sample size. We analyzed 20 studies with 8665 cases and 9953 controls while they only included two studies with 238 cases and 280 controls. For rs4646903 T > C, previous studies showed no association with CRC risk which was consistent with us.

Compared to previous meta-analyses, the present analysis has some advantages. Firstly, we have the largest sample size with the statistic power of 92% to evaluate the associations. Secondly, 90% studies included in our meta-analyses were of high quality score. Thirdly, we conducted sensitivity analysis and found no obvious influence of a single study on the pooled ORs and 95% CIs for rs1048943 A > G. In addition, negative result of publication bias evaluation indicated that our conclusions were unbiased. However, between-study heterogeneity existed, we should draw the conclusion with caution, subgroups analysis of rs1048943 A > G indicated that heterogeneities may be from ethnicity, source of control or controls deviated from HWE. For rs4646903 T > C polymorphism, substantial between-study heterogeneities may originate from ethnicity, source of control and controls deviated from HWE. Besides, for unavailable original data, we failed to analyze clinical features, such as tumor stage, age and sex, etc.

In conclusion, *CYP1A1* rs1048943 A > G polymorphism may increase the CRC risk. However, our study still existed in some limitations, further studies with higher quality and larger sample size are necessary.

## MATERIALS AND METHODS

### Search strategy

We searched publications from PubMed, Web of knowledge, Embase and Chinese database of China National Knowledge Infrastructure (CNKI) and VIP database with the following search items: “CYP1A1” or “Cytochrome P450 1A1” and “polymorphism” or “variant” or “SNP” and “colorectal cancer” or “colon cancer” or “rectal cancer”. The languages were limited to English and Chinese. We updated the search results on January 2016 and confirmed potential relevant studies through the titles and abstracts.

### Selection criteria

All studies included in the meta-analysis are selected according to the following criteria: (a) case-control studies; (b) studies about the associations between *CYP1A1* rs1048943 A > G or rs4646903 T > C and colorectal cancer; (c) studies that contain genotype data; (d) when studies had overlapping populations, the most recent ones with the most complete data set were included. In addition, exclusion criteria were as follows: (a) overlapped articles or studies with overlapping data; (b) review articles, conference reports and dissertations.

### Data extraction

Two investigators (ZXR and ZML) extracted data independently from the eligible studies with the following items: the first author's last name, year of publication, country, ethnicity, source of controls, polymorphism type, number of cases and controls, the frequency of each genotype in cases and controls (Supplementary Table 3), minor allele frequency (MAF) and *p*-value of Hardy Weinberg equilibrium (HWE). To get the accurate data, two investigators discussed together to reach a consensus.

### Quality assessment

We evaluated the quality of the included studies respectively, according to the quality assessment criteria (Supplementary Table 1) [47–50]. The range of quality scores are from 0 (worst) to 15 (best). Publications with quality scores < 10 were categorized as “low quality” and those with quality scores ≥ 10 were categorized as “high quality” [51]. The process of scoring was listed in Supplementary Table 2.

### Statistical analysis

Stata software (version 12.0; Stata Corporation, College Station, TX) was used to perform statistical analyses. We used allelic, heterozygote, homozygote, dominant and recessive as the models. The strength of associations between the *CYP1A1* rs1048943 A > G and rs4646903 T > C and the risk of colorectal cancer were evaluated by the pooled odds ratios (ORs) and 95% confidence intervals (CIs). We set the significance cutoff as a *p*-value of 0.05 for the pooled OR. Between-study heterogeneity was assessed using Chi-squared-based *Q*-test. If heterogeneity *P* value was lower than 0.10, we considered the heterogeneity to be significant and random-effects model was used [52]. Otherwise, the fixed effects model was used [53]. We also performed stratified analyses by ethnicity (Asian and Caucasian), control source (population-based and hospital-based), quality score of studies (low and high) and HWE. Sensitivity analyses were performed to measure the stability of the results by consecutively omitting each study from the meta-analysis (leave-one-out sensitivity analysis). Begg's funnel plot [54] and Egger's test [55] (*P* <0.05 was considered significant) were used to evaluate the publication bias among the literatures.

## SUPPLEMENTARY MATERIALS FIGURES AND TABLES


