# Supplementary material for: Associations between CYP1A1 rs1048943 A > G and rs4646903 T > C genetic variations and colorectal cancer risk: Proof from 26 case-control studies
Source: Oncotarget. 2016 Jun 30;7(32):51365–74. doi: 10.18632/oncotarget.10331 (PMC5239481; doi:10.18632/oncotarget.10331)
Supplement: Supplementary file 1 [file oncotarget-07-51365-s001.pdf]

# Associations between *CYP1A1* rs1048943 A>G and rs4646903 T>C genetic variations and colorectal cancer risk: Proof from 26 case-control studies

## Supplementary Materials

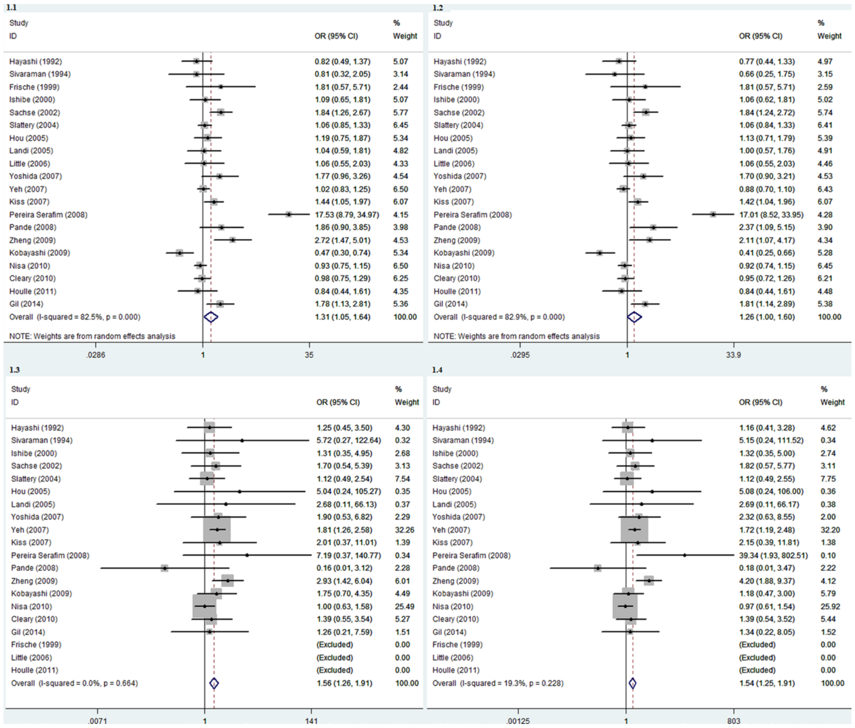


**Supplementary Figure S1: Forest plots of colorectal cancer risk associated with *CYP1A1* rs1048943 A > G polymorphism.** All models showed statistical significance between *CYP1A1* rs1048943 A > G and colorectal cancer risk and the specific values were as follows. **1.1** Dominant model (GG/GA vs. AA), OR = 1.31, 95% CI = 1.05–1.64; **1.2** Heterozygote model (GA vs. AA), OR = 1.26, 95% CI = 1.00–1.60; **1.3** Recessive model (GG vs. GA/AA), OR = 1.56, 95% CI = 1.26–1.91; **1.4** Homozygote model (GG vs. AA), OR = 1.54, 95% CI = 1.25–1.91.

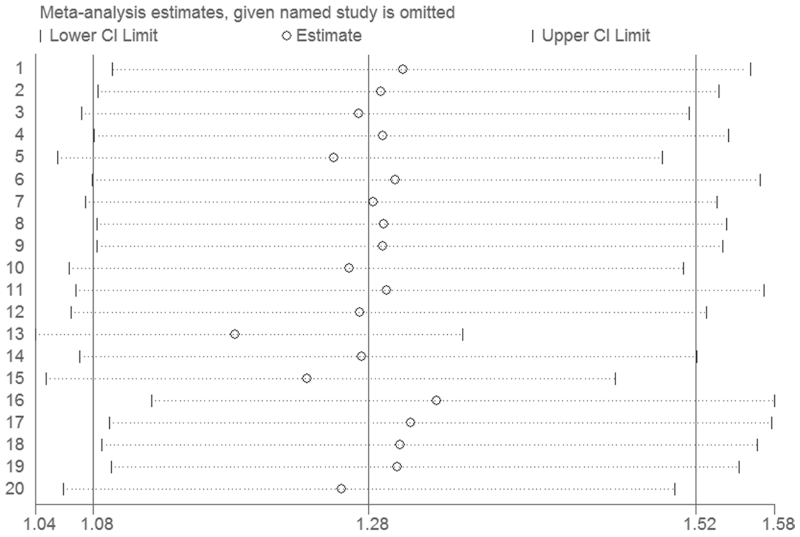


**Supplementary Figure S2: The leave-one-out sensitivity analysis of colorectal cancer risk associated with CYP1A1 rs1048943 A > G polymorphism (G vs. A).**

## Supplementary Table S1: Scale for quality assessment criterion

| Criterion | Score |
|---|---|
| Source of cases<br>Selected from population or cancer registry<br>Selected from hospital<br>Selected from pathology archives, but without description<br>Not described | 3<br>2<br>1<br>0 |
| Source of controls<br>Population –based<br>Blood donors or volunteers<br>Hospital-based (cancer free patients)<br>Not described | 3<br>2<br>1<br>0 |
| Specimens used for determining genotypes<br>White blood cells or normal tissues<br>Tumor tissues or exfoliated cells of tissue | 3<br>0 |
| Hardy-Weinberg equilibrium in controls<br>Hardy- Weinberg equilibrium<br>Hardy- Weinberg disequilibrium | 3<br>0 |
| Total sample size<br>> 1000<br>> 500 and ≤ 1000<br>> 200 and ≤ 500<br>≤ 200 | 3<br>2<br>1<br>0 |

## Supplementary Table S2: The process of scoring on included studies

| Study | Polymorphism type | Source of cases (score) | Source of Controls (score) | Specimens used for determining genotype (score) | HWE in control (score) | Total sample size (score) | score |
|---|---|---|---|---|---|---|---|
| Hayashi,1992 | rs1048943 | 2 | 3 | 3 | 3 | 1 | 12 |
| Sivaraman,1994 | rs1048943 | 2 | 3 | 3 | 3 | 0 | 11 |
| | rs4646903 | 2 | 3 | 3 | 3 | 0 | 11 |
| Frische,1999 | rs1048943 | 3 | 3 | 3 | 3 | 1 | 13 |
| | rs4646903 | 3 | 3 | 3 | 3 | 1 | 13 |
| Ishibe,2000 | rs1048943 | 3 | 3 | 3 | 3 | 1 | 13 |
| | rs4646903 | 3 | 3 | 3 | 3 | 1 | 13 |
| Sachse,2002 | rs1048943 | 2 | 3 | 3 | 0 | 3 | 11 |
| | rs4646903 | 2 | 3 | 3 | 3 | 3 | 14 |
| Ye,2002 | rs4646903 | 2 | 3 | 3 | 3 | 0 | 11 |
| Slattery,2004 | rs1048943 | 3 | 3 | 3 | 0 | 3 | 12 |
| | rs4646903 | 3 | 3 | 3 | 3 | 3 | 15 |
| Hou,2005 | rs1048943 | 2 | 1 | 3 | 3 | 3 | 12 |
| Landi,2005 | rs1048943 | 2 | 1 | 3 | 3 | 2 | 11 |
| | rs4646903 | 2 | 1 | 3 | 3 | 2 | 11 |
| Chen,2005 | rs4646903 | 3 | 3 | 3 | 3 | 1 | 13 |
| Little,2006 | rs1048943 | 3 | 3 | 3 | 3 | 2 | 14 |
| | rs4646903 | 3 | 3 | 3 | 3 | 2 | 14 |
| Bente,2006 | rs4646903 | 2 | 1 | 3 | 3 | 3 | 10 |
| Youshida,2007 | rs1048943 | 2 | 3 | 3 | 3 | 0 | 11 |
| | rs4646903 | 2 | 3 | 3 | 3 | 0 | 11 |
| Yeh,2007 | rs1048943 | 2 | 1 | 3 | 3 | 3 | 12 |
| Kiss,2007 | rs1048943 | 2 | 1 | 3 | 3 | 3 | 12 |
| Pereira, 2008 | rs1048943 | 2 | 3 | 3 | 3 | 1 | 12 |
| Pande,2008 | rs1048943 | 3 | 3 | 3 | 0 | 1 | 10 |
| | rs4646903 | 3 | 3 | 3 | 3 | 1 | 13 |
| Zheng,2009 | rs1048943 | 2 | 3 | 3 | 0 | 0 | 8 |
| | rs4646903 | 2 | 3 | 3 | 3 | 0 | 11 |
| Liu,2009 | rs4646903 | 2 | 1 | 3 | 0 | 0 | 6 |
| Kobayashi,2009 | rs1048943 | 2 | 1 | 3 | 0 | 1 | 7 |
| Nisa,2010 | rs1048943 | 2 | 3 | 3 | 3 | 3 | 14 |
| | rs4646903 | 2 | 3 | 3 | 3 | 3 | 14 |
| Cleary,2010 | rs1048943 | 2 | 3 | 3 | 0 | 3 | 11 |
| | rs4646903 | 2 | 3 | 3 | 3 | 3 | 14 |
| Houlle,2011 | rs1048943 | 2 | 1 | 3 | 3 | 2 | 11 |
| | rs4646903 | 2 | 1 | 3 | 3 | 2 | 11 |
| Darazy,2011 | rs4646903 | 2 | 3 | 0 | 0 | 0 | 5 |
| Saeed,2013 | rs4646903 | 3 | 1 | 3 | 3 | 0 | 10 |
| Gil,2014 | rs1048943 | 2 | 1 | 3 | 3 | 2 | 11 |

HWE, Hardy-Weinberg equilibrium.

## Supplementary Table 3: The genotype distribution of included studies

### Supplementary Table 3.1: The genotype distribution of rs1048943 A > G polymorphism of included studies

| | case | | | | Control | | | |
|---|---|---|---|---|---|---|---|---|
| **Study** | **Case sample** | **AA (*n*/%)** | **GA (*n*/%)** | **GG (*n*/%)** | **Control sample** | **AA (*n*/%)** | **GA (*n*/%)** | **GG (*n*/%)** |
| Hayashi,1992 | 85 | 59 (69.41) | 21 (24.71) | 5 (5.88) | 358 | 233 (65.08) | 108 (30.17) | 17 (4.75) |
| Sivaraman,1994 | 43 | 32 (74.42) | 9 (20.93) | 2 (4.65) | 47 | 33 (70.21) | 14 (29.79) | 0 (0.00) |
| Frische,1999 | 187 | 174 (93.05) | 13 (6.95) | 0 (0.00) | 101 | 97 (96.04) | 4 (3.96) | 0 (0.00) |
| Ishibe,2000 | 212 | 176 (83.02) | 31 (14.62) | 5 (2.36) | 221 | 186 (84.16) | 31 (14.03) | 4 (1.81) |
| Sachse,2002 | 490 | 415 (84.69) | 68 (13.88) | 7 (1.43) | 592 | 539 (91.05) | 48 (8.11) | 5 (0.84) |
| Slattery,2004 | 1791 | 1632 (91.12) | 148 (8.26) | 11 (0.61) | 2180 | 1997 (91.61) | 171 (7.84) | 12 (0.55) |
| Hou,2005 | 675 | 633 (93.78) | 40 (5.93) | 2 (0.30) | 679 | 643 (94.70) | 36 (5.30) | 0 (0.00) |
| Landi,2005 | 362 | 333 (91.99) | 28 (7.73) | 1 (0.28) | 323 | 298 (92.26) | 25 (7.74) | 0 (0.00) |
| Little,2006 | 251 | 235 (93.63) | 16 (6.37) | 0 (0.00) | 396 | 372 (93.94) | 24 (6.06) | 0 (0.00) |
| Youshida,2007 | 66 | 34 (51.52) | 27 (40.91) | 5 (7.58) | 212 | 79 (65.29) | 37 (30.58) | 5 (4.13) |
| Yeh,2007 | 717 | 400 (55.79) | 228 (31.80) | 89 (12.41) | 729 | 410 (56.24) | 266 (36.49) | 53 (7.27) |
| Kiss,2007 | 500 | 386 (77.20) | 110 (22.00) | 4 (0.80) | 500 | 415 (83.00) | 83 (16.60) | 2 (0.40) |
| Pereira, 2008 | 114 | 14 (12.28) | 97 (85.09) | 3 (2.63) | 114 | 81 (71.05) | 33 (28.95) | 0 (0.00) |
| ()Pande,2008 | 120 | 99 (82.50) | 21 (17.50) | 0 (0.00) | 137 | 123 (89.78) | 11 (8.03) | 3 (2.19) |
| Zheng,2009 | 79 | 23 (29.11) | 31 (39.24) | 25 (31.65) | 110 | 58 (52.73) | 37 (33.64) | 15 (13.64) |
| Kobayashi,2009 | 105 | 65 (61.90) | 32 (30.48) | 8 (7.62) | 289 | 125 (43.25) | 151 (52.25) | 13(4.50) |
| Nisa,2010 | 685 | 418 (61.02) | 231 (33.72) | 36 (5.26) | 78 | 461 (59.25) | 276 (35.48) | 41 (5.27) |
| Cleary,2010 | 1160 | 1052 (90.69) | 98 (8.45) | 10 (0.86) | 1288 | 1166 (90.53) | 114 (8.85) | 8 (0.62) |
| Houlle,2011 | 329 | 313 (95.14) | 16 (4.86) | 0 (0.00) | 419 | 395 (94.27) | 24 (5.73) | 0 (0.00) |
| Gil,2014 | 476 | 414 (86.97) | 59 (12.39) | 3 (0.63) | 400 | 369 (92.25) | 29 (7.25) | 2 (0.50) |

### Supplementary Table 3.2: The genotype distribution of rs4646903 T > C polymorphism of included studies

| | case | | | | Control | | | |
|---|---|---|---|---|---|---|---|---|
| **Study** | **Case sample** | **TT (*n*/%)** | **CT (*n*/%)** | **CC (*n*/%)** | **Control sample** | **TT (*n*/%)** | **CT (*n*/%)** | **CC (*n*/%)** |
| Sivaraman,1994 | 43 | 23 (53.49) | 10 (23.26) | 10 (23.26) | 47 | 33 (70.21) | 14 (29.79) | 0 (0.00) |
| Frische,1999 | 187 | 151 (80.75) | 35 (18.72) | 1 (0.53) | 101 | 90 (89.11) | 11 (10.89) | 0 (0.00) |
| Ishibe,2000 | 211 | 170 (80.57) | 37 (17.54) | 4 (1.90) | 221 | 169 (76.47) | 47 (21.27) | 5 (2.26) |
| Ye,2002 | 41 | 35 (85.37) | 6 (14.63) | 0 (0.00) | 82 | 73 (89.02) | 9 (10.98) | 0 (0.00) |
| Sachse,2002 | 490 | 389 (79.39) | 90 (18.37) | 11 (2.24) | 592 | 476 (80.41) | 111 (18.75) | 5 (0.84) |
| Slattery,2004 | 1805 | 1380 (76.45) | 387 (21.44) | 38 (2.11) | 2164 | 1639 (75.74) | 479 (22.13) | 46 (2.13) |
| Landi,2005 | 358 | 289 (80.73) | 66 (18.44) | 3 (0.84) | 305 | 235 (77.05) | 66 (21.64) | 4 (1.31) |
| Chen,2005 | 139 | 65 (46.76) | 60 (43.17) | 14 (10.07) | 340 | 122 (35.88) | 165 (48.53) | 53 (15.59) |
| Bente,2006 | 118 | 94 (79.66) | 20 (16.95) | 4 (3.39) | 100 | 91 (91.00) | 9 (9.00) | 0 (0.00) |
| Little,2006 | 232 | 190 (81.90) | 38 (16.38) | 4 (1.72) | 378 | 310 (82.01) | 66 (17.46) | 2 (0.53) |
| Youshida,2007 | 66 | 20 (30.30) | 36 (54.55) | 10 (15.15) | 121 | 49 (40.50) | 54 (44.63) | 18 (14.88) |
| Pande,2008 | 120 | 83 (69.17) | 37 (30.83) | 0 (0.00) | 137 | 104 (75.91) | 29 (21.17) | 4 (2.92) |
| Liu,2009 | 75 | 38 (50.67) | 19 (25.33) | 18 (24.00) | 100 | 65 (62.00) | 21 (21.00) | 14 (14.00) |
| Zheng,2009 | 79 | 35 (44.30) | 33 (41.77) | 11 (13.92) | 110 | 57 (51.82) | 41 (37.27) | 12 (10.91) |
| Nisa,2010 | 685 | 283 (41.31) | 307 (44.28) | 95 (13.87) | 778 | 305 (39.20) | 368 (47.30) | 105 (13.50) |
| Cleary,2010 | 1163 | 904 (77.73) | 247 (21.24) | 12 (1.03) | 1290 | 1004 (77.83) | 260 (20.16) | 26 (2.02) |
| Darazy,2011 | 46 | 42 (91.30) | 2 (4.35) | 2 (4.35) | 56 | 54 (96.43) | 1 (1.79) | 1 (1.79) |
| Houlle,2011 | 329 | 265 (80.55) | 61 (18.54) | 3 (0.91) | 419 | 333 (79.47) | 79 (18.85) | 7 (1.67) |
| Saeed,2013 | 94 | 70 (74.47) | 21 (22.34) | 3 (3.19) | 79 | 73 (92.41) | 6 (7.59) | 0 (0.00) |
